# Supplementary material for: Importance of small vessel disease as a possible cause of sudden sensorineural hearing loss
Source: PLoS One. 2024 May 7;19(5):e0302447. doi: 10.1371/journal.pone.0302447 (PMC11075872; doi:10.1371/journal.pone.0302447)
Supplement: S1 Table — (PDF) [file pone.0302447.s001.pdf]

S1 Table. Classification codes of anti-hypertensive drugs

Ingredient name / Main ingredient code					
Alacepril 25 mg	104201ATB	Valsartan 40 mg	247103ATB	Fimasartan potassium trihydrate (as fimasartan potassium 0.12 g)	515202ATB
Alacepril 50 mg	104202ATB	Valsartan 0.32 g	247104ATB	Fimasartan potassium trihydrate (as fimasartan potassium 30 mg)	515203ATB
Amlodipine besylate (as amlodipine 5 mg)	107601ATB	Chlorthalidone 12.5 mg	262100ATB	Olmesartan medoxomil 40 mg	519700ATB
Amlodipine besylate (as amlodipine 10 mg)	107602ATB	Metoprolol succinate 47.5 mg	262400ATR	Olmesartan medoxomil 20 mg	519800ATB
Amlodipine besylate (as amlodipine 2.5 mg)	107603ATB	Losartan potassium (as losartan 45.8 mg)	262500ATB	Olmesartan medoxomil 40 mg	520000ATB
Atenolol 25 mg	111402ATB	Losartan potassium (as losartan 91.6 mg)	378900ATB	Telmisartan 40 mg	521200ATB
Atenolol 50 mg	111403ATB	Losartan potassium (as losartan 91.6 mg)	486900ATB	Telmisartan 40 mg	521300ATB
Barnidipine hydrochloride 10 mg	114001ACH	Spironolactone 25 mg	262700ATB	Telmisartan 80 mg	521400ATB
Barnidipine hydrochloride 15 mg	114002ACH	Valsartan 80 mg	356400ATB	S-amlodipine besylate (as S-amlodipine 5 mg)	644800ATB
Barnidipine hydrochloride 5 mg	114003ACH	Valsartan 0.16 g	442600ATB	Hydrochlorothiazide 12.5 mg	522000ATB
Benidipine hydrochloride 2 mg	115101ATB	Telmisartan 40 mg	378801ATB	Hydrochlorothiazide 12.5 mg	526800ATB
Benidipine hydrochloride 4 mg	115102ATB	Telmisartan 80 mg	378802ATB	Valsartan 80 mg	522200ATB
Benidipine hydrochloride 8 mg	115103ATB	Irbesartan 0.15 g	385700ATB	Valsartan 0.16 g	522300ATB
Benidipine hydrochloride 6 mg	115104ATB	Irbesartan 0.3 g	385800ATB	Valsartan 0.16 g	522400ATB
Betaxolol hydrochloride 10 mg	116801ATB	Hydrochlorothiazide 12.5 mg	423700ATB	Valsartan 80 mg	522600ATB
Betaxolol hydrochloride 20 mg	116803ATB	Eprosartan mesylate (as eprosartan 0.6 g)	429201ATB	Valsartan 0.16 g	522700ATB
Bevantolol hydrochloride 0.1 g	117001ATB	Telmisartan 40 mg	443200ATB	Valsartan 0.16 g	522800ATB
Bevantolol hydrochloride 50 mg	117002ATB	Telmisartan 80 mg	443300ATB	Valsartan 80 mg	522900ATB
Candesartan cilexetil 8 mg	122601ATB	Telmisartan 80 mg	502600ATB	Valsartan 0.16 g	523000ATB

Candesartan cilexetil 16 mg	122602ATB	Ramipril 2.5 mg	447100ATB	Valsartan 0.16 g	523100ATB
Candesartan cilexetil 32 mg	122603ATB	Ramipril 5 mg	447200ATB	Valsartan 80 mg	523200ATB
Captopril 12.5 mg	122901ATB	Amlodipine maleate (as amlodipine 5 mg)	459801ACH	Valsartan 0.16 g	523300ATB
Captopril 25 mg	122902ATB	Amlodipine maleate (as amlodipine 5 mg)	459801ATB	Valsartan 0.16 g	523400ATB
Captopril 50 mg	122903ATB	Amlodipine maleate (as amlodipine 2.5 mg)	459802ACH	Amlodipine maleate (as amlodipine 10 mg)	547500ATB
Carvedilol 12.5 mg	125001ATB	Amlodipine camsylate (as amlodipine 5 mg)	459901ATB	Amlodipine maleate (as amlodipine 5 mg)	547600ATB
Carvedilol 25 mg	125002ATB	Hydrochlorothiazide 12.5 mg	460500ATB	Amlodipine maleate (as amlodipine 5 mg)	547700ATB
Carvedilol 6.25 mg	125003ATB	Amlodipine adipate (as amlodipine 5 mg)	464601ATB	Olmesartan medoxomil 20 mg	547800ATB
Carvedilol 64 mg	125004ACR	Olmesartan medoxomil 20 mg	468501ATB	Olmesartan medoxomil 40 mg	547900ATB
Carvedilol 3.125 mg	125005ATB	Olmesartan medoxomil 10 mg	468502ATB	Olmesartan medoxomil 40 mg	548000ATB
Carvedilol 32 mg	125006ACR	Olmesartan medoxomil 40 mg	468503ATB	Olmesartan medoxomil 20 mg	631300ATB
Carvedilol 16 mg	125007ACR	Hydrochlorothiazide 6.25 mg	469800ATB	Sildenafil citrate (as sildenafil 20 mg)	553301ATB
Carvedilol 8 mg	125008ACR	Hydrochlorothiazide 6.25 mg	469900ATB	Perindopril arginine 5 mg	556200ATB
Cilazapril 0.5 mg	133001ATB	Hydrochlorothiazide 6.25 mg	470000ATB	Ambrisentan 5 mg	564701ATB
Cilazapril 1 mg	133002ATB	Amlodipine mesylate monohydrate (as amlodipine 5 mg)	470801ATB	Ambrisentan 10 mg	564702ATB
Cilazapril 2.5 mg	133003ATB	Amlodipine mesylate monohydrate (as amlodipine 10 mg)	470802ATB	Olmesartan medoxomil 40 mg	629400ATB
Cilnidipine 10 mg	133101ATB	Amlodipine orotate (as amlodipine 5 mg)	476201ATB	Olmesartan medoxomil 20 mg	629500ATB
Cilnidipine 5 mg	133102ATB	Amlodipine nicotinate (as amlodipine 5 mg)	479701ATB	Olmesartan medoxomil 40 mg	629600ATB
Doxazosin mesylate (as doxazocin 1 mg)	149101ATB	S-atenolol 25 mg	483101ATB	Micronized macitentan 10 mg	632201ATB
Doxazosin mesylate (as doxazocin 2 mg)	149102ATB	S-atenolol 12.5 mg	483102ATB	S-amlodipine besylate (as S-amlodipine 2.5 mg)	632800ATB
Doxazosin mesylate (as doxazocin 4 mg)	149104ATR	S-amlodipine besylate (as S-amlodipine 2.5 mg)	483201ATB	S-amlodipine besylate (as S-amlodipine 2.5 mg)	632900ATB

Enalapril maleate 10 mg	151601ATB	S-amlodipine besylate (as S-amlodipine 5 mg)	483202ATB	S-amlodipine besylate (as S-amlodipine 5 mg)	633000ATB
Enalapril maleate 5 mg	151603ATB	Phenoxybenzamine 10 mg	483401ACH	Losartan potassium (as losartan 45.8 mg)	637400ATB
Felodipine 5 mg	157501ATR	Bosentan hydrate (as bosentan 62.5 mg)	485201ATB	Losartan potassium (as losartan 45.8 mg)	637500ATB
Felodipine 2.5 mg	157503ATR	S-amlodipine nicotinate (as S-amlodipine 2.5 mg)	486501ATB	Losartan potassium (as losartan 91.6 mg)	637600ATB
Hydralazine hydrochloride 25 mg	170701ATB	S-amlodipine nicotinate (as S-amlodipine 5 mg)	486502ATB	Fimasartan potassium trihydrate (as fimasartan potassium 30 mg)	651900ATB
Imidapril hydrochloride 10 mg	173401ATB	Nebivolol hydrochloride (as nebivolol 5 mg)	489501ATB	Fimasartan potassium trihydrate (as fimasartan potassium 30 mg)	652000ATB
Imidapril hydrochloride 5 mg	173402ATB	Nebivolol 2.5 mg	489502ATB	Fimasartan potassium trihydrate (as fimasartan potassium 60 mg)	652100ATB
Indapamide 1.5 mg	174401ATR	Nebivolol hydrochloride (as nebivolol 1.25 mg)	489503ATB	Amlodipine besylate (as amlodipine 5 mg)	652700ATB
Irbesartan 0.15 g	177301ATB	Valsartan 0.16 g	492800ATB	Selexipag 0.2 mg	652301ATB
Irbesartan 0.3 g	177303ATB	Valsartan 80 mg	492900ATB	Selexipag 0.8 mg	652302ATB
Lacidipine 2 mg	180301ATB	Valsartan 0.16 g	495800ATB	Selexipag 0.4 mg	652303ATB
Lacidipine 4 mg	180302ATB	Olmesartan medoxomil 20 mg	500500ATB	Candesartan cilexetil 8 mg	652900ATB
Lacidipine 6 mg	180303ATB	Olmesartan medoxomil 40 mg	582200ATB	Candesartan cilexetil 16 mg	653000ATB
Lercanidipine hydrochloride 10 mg	182001ATB	Olmesartan medoxomil 40 mg	582400ATB	Candesartan cilexetil 16 mg	653100ATB
Lisinopril 10 mg	184501ATB	Perindopril arginine 5 mg	501601ATB	S-carvedilol 6.25 mg	662201ATB
Losartan potassium (as losartan 45.8 mg)	185701ATB	Perindopril arginine 10 mg	501602ATB	S-carvedilol 12.5 mg	662202ATB
Losartan potassium (as losartan 91.6 mg)	185702ATB	Losartan potassium (as losartan 91.6 mg)	502700ATB	Azilsartan medoxomil potassium (as azilsartan medoxomil 20 mg)	662401ATB
Manidipine hydrochloride 10 mg	188001ATB	Losartan potassium (as losartan 45.8 mg)	503000ATB	Azilsartan medoxomil potassium (as azilsartan medoxomil 80 mg)	662402ATB

Manidipine hydrochloride 20 mg	188002ATB	Losartan potassium (as losartan 45.8 mg)	513900ATB	Azilsartan medoxomil potassium (as azilsartan medoxomil 40 mg)	662403ATB
Minoxidil 5 mg	196102ATB	Zofenopril calcium 7.5 mg	510401ATB	Chlorthalidone 12.5 mg	662800ATB
Nicardipine hydrochloride 40 mg	201003ACR	Zofenopril calcium 15 mg	510402ATB	Chlorthalidone 12.5 mg	662900ATB
Perindopril tertbutylamine 4 mg	211301ATB	Zofenopril calcium 30 mg	510403ATB	Chlorthalidone 25 mg	663000ATB
Perindopril tertbutylamine 8 mg	211302ATB	Telmisartan 80 mg	511500ATB	Telmisartan 40 mg	663500ATB
Ramipril 2.5 mg	222401ATB	Telmisartan 40 mg	511600ATB	Telmisartan 80 mg	663600ATB
Ramipril 5 mg	222402ATB	Telmisartan 40 mg	511700ATB	Telmisartan 80 mg	663700ATB
Ramipril 10 mg	222404ATB	Telmisartan 80 mg	623100ATB	Telmisartan 80 mg	663800ATB
Temocapril hydrochloride 2 mg	235002ATB	Olmesartan medoxomil 20 mg	513600ATB	Chlorthalidone 12.5 mg	673500ATB
Valsartan 80 mg	247101ATB	Fimasartan potassium trihydrate (as fimasartan potassium 60 mg)	515201ATB	Chlorthalidone 25 mg	673600ATB
Valsartan 0.16 g	247102ATB				
