# Supplementary material for: Importance of small vessel disease as a possible cause of sudden sensorineural hearing loss
Source: PLoS One. 2024 May 7;19(5):e0302447. doi: 10.1371/journal.pone.0302447 (PMC11075872; doi:10.1371/journal.pone.0302447)
Supplement: S2 Table — (PDF) [file pone.0302447.s002.pdf]

S2 Table. Classification codes of diabetes mellitus drugs

Ingredient name / Main ingredient code / Classification					
Acarbose 100 mg	100601ATB	AGI	Exenatide 0.60 mg/2.4 mL	512131BIJ	GLP-1
Acarbose 50 mg	100602ATB	AGI	Sitagliptin 50 mg	513700ATB	DPP-4
Glibenclamide 5 mg	165402ATB	Sulfonylurea	Sitagliptin 50 mg	513700ATR	DPP-4
Gliclazide 40 mg	165601ACS	Sulfonylurea	Saxagliptin 5 mg	518500ATR	DPP-4
Gliclazide 80 mg	165602ACS	Sulfonylurea	Saxagliptin 5 mg	518600ATR	DPP-4
Gliclazide 80 mg	165602ATB	Sulfonylurea	Mitiglinide 10 mg	518800ATB	Meglitinides
Gliclazide 30 mg	165603ATR	Sulfonylurea	Vildagliptin 50 mg	519600ATB	DPP-4
Gliclazide 60 mg	165604ATR	Sulfonylurea	Linagliptin 2.5 mg	520500ATB	DPP-4
Glimepiride 1 mg	165701ATB	Sulfonylurea	Linagliptin 2.5 mg	520600ATB	DPP-4
Glimepiride 2 mg	165702ATB	Sulfonylurea	Linagliptin 2.5 mg	520700ATB	DPP-4
Glimepiride 3 mg	165703ATB	Sulfonylurea	Voglibose 0.2 mg	523600ATB	AGI
Glimepiride 4 mg	165704ATB	Sulfonylurea	Voglibose 0.2 mg	523700ATB	AGI
Glipizide 5 mg	165801ATB	Sulfonylurea	Gemigliptin 25 mg	523800ATR	DPP-4
Gliquidone 30 mg	165901ATB	Sulfonylurea	Sitagliptin 100 mg	524700ATR	DPP-4
Insulin 100 IU/mL, 10 mL (human insulin)	170101BIJ	Insulin	Pioglitazone 30 mg	525500ATB	TZD
Insulin 100 IU/mL, 3 mL (human insulin)	170102BIJ	Insulin	Pioglitazone 30 mg	525600ATB	TZD
Insulin 40 IU/mL, 10 mL (human insulin)	170103BIJ	Insulin	Lobeglitazone 0.5 mg	525901ATB	TZD
Insulin 100 IU/mL, 3 mL (human insulin)	170130BIJ	Insulin	Dapagliflozin 5 mg	527301ATB	SGLT-2
Insulin 100 IU/mL, 10 mL (human insulin)	170131BIJ	Insulin	Dapagliflozin 10 mg	527302ATB	SGLT-2
Insulin 100 IU/mL, 10 mL (N70/R30)	170401BIJ	Insulin	Saxagliptin 2.5 mg	613301ATB	DPP-4
Insulin 100 IU/mL, 3 mL (N70/R30)	170402BIJ	Insulin	Saxagliptin 5 mg	613302ATB	DPP-4
Insulin 100 IU/mL, 3 mL (N70/R30)	170430BIJ	Insulin	Linagliptin 5 mg	616401ATB	DPP-4
Insulin 100 IU/mL, 10 mL (N70/R30)	170431BIJ	Insulin	Gemigliptin 50 mg	619101ATB	DPP-4
Insulin 100 IU/mL, 3 mL (N80/R20)	170502BIJ	Insulin	Alogliptin 6.25 mg	624201ATB	DPP-4
Insulin 100 IU/mL, 10 mL (insulin lispro)	175301BIJ	Insulin	Alogliptin 12.5 mg	624202ATB	DPP-4

Insulin 100 IU/mL, 3 mL (insulin lispro)	175302BIJ	Insulin	Alogliptin 25 mg	624203ATB	DPP-4
Insulin 100 IU/mL, 3 mL (insulin lispro protamine 75/insulin lispro 25)	175304BIJ	Insulin	Lixisenatide 0.1 mg/mL, 3 mL	626601BIJ	GLP-1
Insulin 100 IU/mL, 3 mL (insulin lispro)	175330BIJ	Insulin	Lixisenatide 0.05 mg/mL, 3 mL	626602BIJ	GLP-1
Insulin 100 IU/mL, 10 mL (insulin lispro)	175331BIJ	Insulin	Lixisenatide 0.05 mg/mL, 3 mL	626630BIJ	GLP-1
Insulin 100 IU/mL, 3 mL (insulin lispro protamine 75/insulin lispro 25)	175332BIJ	Insulin	Lixisenatide 0.1 mg/mL, 3 mL	626631BIJ	GLP-1
Insulin 100 IU/mL, 3 mL (insulin lispro protamine 50/insulin lispro 50)	175333BIJ	Insulin	Insulin 100 IU/mL, 3 mL (insulin degludec 70/insulin aspart 30)	626700BIJ	Insulin
Metformin 250 mg	191501ATB	Metformin	Insulin 100 IU/mL, 3 mL (insulin degludec)	626801BIJ	Insulin
Metformin 500 mg	191502ATB	Metformin	Insulin 200 IU/mL, 3 mL (insulin degludec)	626802BIJ	Insulin
Metformin 500 mg	191502ATR	Metformin	Insulin 100 IU/mL, 3 mL (insulin degludec)	626830BIJ	Insulin
Metformin 850 mg	191503ATB	Metformin	Insulin 200 IU/mL, 3 mL (insulin degludec)	626831BIJ	Insulin
Metformin 1,000 mg	191504ATB	Metformin	Teneligliptin 20 mg	627301ATB	DPP-4
Metformin 1,000 mg	191504ATR	Metformin	Empagliflozin 10 mg	628201ATB	SGLT-2
Metformin 750 mg	191505ATR	Metformin	Empagliflozin 25 mg	628202ATB	SGLT-2
Voglibose 0.2 mg	249001ATB	AGI	Alogliptin 12.5 mg	630300ATB	DPP-4
Voglibose 0.2 mg	249001ATD	AGI	Alogliptin 12.5 mg	630400ATB	DPP-4
Voglibose 0.3 mg	249002ATB	AGI	Alogliptin 25 mg	630500ATB	DPP-4
Voglibose 0.3 mg	249002ATD	AGI	Alogliptin 25 mg	630600ATB	DPP-4
Rosiglitazone 4 mg	348002ATB	TZD	Nateglinide 120 mg	631900ATB	Meglitinides
Repaglinide 0.5 mg	379501ATB	Meglitinides	Gemigliptin 50 mg	632000ATR	DPP-4
Repaglinide 1 mg	379502ATB	Meglitinides	Repaglinide 2 mg	632100ATB	Meglitinides
Repaglinide 2 mg	379503ATB	Meglitinides	Alogliptin 12.5 mg	635600ATB	DPP-4
Miglitol 50 mg	406201ATB	AGI	Alogliptin 12.5 mg	635700ATB	DPP-4
Miglitol 100 mg	406202ATB	AGI	Ipragliflozin 50 mg	636101ATB	SGLT-2
Glibenclamide 2.5 mg	421100ATB	Sulfonylurea	Repaglinide 1 mg	637200ATB	Meglitinides
Nateglinide 30 mg	430201ATB	Meglitinides	Anagliptin 100 mg	639601ATB	DPP-4

Nateglinide 90 mg	430202ATB	Meglitinides	Dulaglutide 0.75 mg/0.5 mL, 0.5 mL	639701BIJ	GLP-1
Nateglinide 120 mg	430203ATB	Meglitinides	Dulaglutide 1.5 mg/0.5 mL, 0.5 mL	639702BIJ	GLP-1
Pioglitazone 15 mg	431901ATB	TZD	Dapagliflozin 10 mg	639800ATR	SGLT-2
Pioglitazone 30 mg	431902ATB	TZD	Dapagliflozin 10 mg	641400ATR	SGLT-2
Insulin 100 IU/mL, 3 mL (insulin aspart)	441301BIJ	Insulin	Teneligliptin 10 mg	641800ATR	DPP-4
Insulin 100 IU/mL, 10 mL (insulin aspart)	441302BIJ	Insulin	Teneligliptin 10 mg	641900ATR	DPP-4
Insulin 100 IU/mL, 3 mL (insulin aspart protamine 70/insulin aspart 30)	441303BIJ	Insulin	Teneligliptin 20 mg	642000ATR	DPP-4
Insulin 100 IU/mL, 3 mL (insulin aspart protamine 70/insulin aspart 30)	441304BIJ	Insulin	Albiglutide 30 mg	644501BIJ	GLP-1
Insulin 100 IU/mL, 3 mL (insulin aspart protamine 50/insulin aspart 50)	441305BIJ	Insulin	Albiglutide 50 mg	644502BIJ	GLP-1
Insulin 100 IU/mL, 3 mL (insulin aspart)	441330BIJ	Insulin	Nateglinide 90 mg	644900ATB	Meglitinides
Insulin 100 IU/mL, 10 mL (insulin aspart)	441331BIJ	Insulin	Gemigliptin 50 mg	645000ATR	DPP-4
Insulin 100 IU/mL, 3 mL (insulin aspart protamine 70/insulin aspart 30)	441332BIJ	Insulin	Evogliptin 5 mg	645301ATB	DPP-4
Insulin 100 IU/mL, 3 mL (insulin aspart protamine 50/insulin aspart 50)	441333BIJ	Insulin	Anagliptin 100 mg	648400ATB	DPP-4
Insulin 100 IU/mL, 3 mL (insulin aspart protamine 70/insulin aspart 30)	441334BIJ	Insulin	Anagliptin 100 mg	648500ATB	DPP-4
Glibenclamide 2.5 mg	443400ATB	Sulfonylurea	Anagliptin 100 mg	648600ATB	DPP-4
Glibenclamide 5 mg	443500ATB	Sulfonylurea	Empagliflozin 5 mg	649000ATB	SGLT-2
Insulin 100 IU/mL, 3 mL (insulin glargine)	461801BIJ	Insulin	Empagliflozin 12.5 mg	649100ATB	SGLT-2
Insulin 100 IU/mL, 10 mL (insulin glargine)	461802BIJ	Insulin	Empagliflozin 12.5 mg	649200ATB	SGLT-2
Insulin 300 IU/mL, 1.5 mL (insulin glargine)	461804BIJ	Insulin	Empagliflozin 12.5 mg	649300ATB	SGLT-2
Insulin 100 IU/mL, 3 mL (insulin glargine)	461830BIJ	Insulin	Empagliflozin 5 mg	649400ATB	SGLT-2
Insulin 100 IU/mL, 10 mL (insulin glargine)	461831BIJ	Insulin	Empagliflozin 5 mg	649500ATB	SGLT-2
Insulin 300 IU/mL, 1.5 mL (insulin glargine)	461832BIJ	Insulin	Evogliptin 5 mg	649900ATR	DPP-4
Glibenclamide 1.25 mg	471900ATB	Sulfonylurea	Evogliptin 2.5 mg	650000ATR	DPP-4
Glimepiride 1 mg	474200ATB	Sulfonylurea	Evogliptin 2.5 mg	650100ATR	DPP-4

Glimepiride 2 mg	474300ATB	Sulfonylurea	Lobeglitazone 0.25 mg	653800ATR	TZD
Glimepiride 2 mg	474300ATR	Sulfonylurea	Lobeglitazone 0.25 mg	653900ATR	TZD
Insulin 100 IU/mL, 3 mL (insulin glulisine)	484901BIJ	Insulin	Lobeglitazone 0.5 mg	654000ATR	TZD
Insulin 100 IU/mL, 10 mL (insulin glulisine)	484902BIJ	Insulin	Gemigliptin 25 mg	654100ATR	DPP-4
Insulin 100 IU/mL, 3 mL (insulin glulisine)	484930BIJ	Insulin	Lobeglitazone 0.25 mg	655700ATR	TZD
Insulin 100 IU/mL, 10 mL (insulin glulisine)	484931BIJ	Insulin	Gemigliptin 50 mg	664600ATB	DPP-4
Mitiglinide 10 mg	486101ATB	Meglitinides	Gemigliptin 50 mg	664700ATB	DPP-4
Insulin 100 IU/mL, 3 mL (insulin detemir)	488701BIJ	Insulin	Gemigliptin 50 mg	664800ATB	DPP-4
Insulin 100 IU/mL, 3 mL (insulin detemir)	488730BIJ	Insulin	Lixisenatide 0.033 mcg/mL, 3 mL	666700BIJ	GLP-1
Gliclazide 80 mg	497200ATB	Sulfonylurea	Lixisenatide 0.05 mcg/mL, 3 mL	667000BIJ	GLP-1
Pioglitazone 15 mg	498100ATB	TZD	Metformin 500 mg	671800ATR	Metformin
Glimepiride 1 mg	498600ATB	Sulfonylurea	Metformin 750 mg	671900ATR	Metformin
Vildagliptin 50 mg	500801ATB	DPP-4	Metformin 500 mg	672000ATR	Metformin
Sitagliptin 25 mg	501101ATB	DPP-4	Metformin 750 mg	672100ATR	Metformin
Sitagliptin 50 mg	501102ATB	DPP-4	Metformin 500 mg	672500ATR	Metformin
Sitagliptin 100 mg	501103ATB	DPP-4	Metformin 750 mg	672600ATR	Metformin
Sitagliptin 50 mg	502300ATB	DPP-4	Metformin 500 mg	672700ATR	Metformin
Sitagliptin 50 mg	502300ATR	DPP-4	Metformin 750 mg	672800ATR	Metformin
Sitagliptin 50 mg	502900ATB	DPP-4	Metformin 500 mg	672900ATR	Metformin
Vildagliptin 50 mg	507000ATB	DPP-4	Metformin 750 mg	673000ATR	Metformin
Vildagliptin 50 mg	507100ATB	DPP-4	Metformin 1,000 mg	673800ATR	Metformin
Exenatide 0.30 mg/1.2 mL	512101BIJ	GLP-1	Ertugliflozin 5 mg	674301ATB	SGLT-2
Exenatide 0.60 mg/2.4 mL	512102BIJ	GLP-1	Ertugliflozin 15 mg	674302ATB	SGLT-2
Exenatide 0.30 mg/1.2 mL	512130BIJ	GLP-1			
Insulin 100 IU/mL, 3 mL (insulin lispro protamine 50/insulin lispro 50)				507401BIJ	Insulin
