# Supplementary material for: Importance of small vessel disease as a possible cause of sudden sensorineural hearing loss
Source: PLoS One. 2024 May 7;19(5):e0302447. doi: 10.1371/journal.pone.0302447 (PMC11075872; doi:10.1371/journal.pone.0302447)
Supplement: S3 Table — (PDF) [file pone.0302447.s003.pdf]

S3 Table. Classification codes of Dyslipidemia drugs

Ingredient name / Main ingredient code / Classification					
simvastatin	227803AT B	Statin	rosuvastatin and valsartan	525200ATB	Statin
simvastatin	227801AT B	Statin	rosuvastatin and valsartan	629700ATB	Statin
simvastatin	227801AT R	Statin	rosuvastatin and valsartan	629800ATB	Statin
simvastatin	227802AT B	Statin	rosuvastatin and olmesartan	644100ATB	Statin
simvastatin	227802AT D	Statin	rosuvastatin and olmesartan	526300ATB	Statin
simvastatin	227806AT B	Statin	rosuvastatin and olmesartan	526400ATB	Statin
simvastatin	227805AT B	Statin	rosuvastatin and olmesartan	526900ATB	Statin
lovastatin	185801AT B	Statin	rosuvastatin and olmesartan	526500ATB	Statin
lovastatin	185901AT B	Statin	rosuvastatin and olmesartan	644200ATB	Statin
pravastatin	216601AT B	Statin	atorvastatin and irbesartan	524100ATB	Statin
pravastatin	216603AT B	Statin	atorvastatin and irbesartan	524000ATB	Statin
pravastatin	216604AT B	Statin	atorvastatin and irbesartan	527100ATB	Statin
pravastatin	216602AT B	Statin	atorvastatin and irbesartan	527000ATB	Statin
fluvastatin	162401AC H	Statin	atorvastatin and irbesartan	635100ATB	Statin
fluvastatin	162402AC H	Statin	atorvastatin and irbesartan	635200ATB	Statin
fluvastatin	162403AT R	Statin	pitavastatin and valsartan	634900ATB	Statin
atorvastatin	111501AT B	Statin	pitavastatin and valsartan	635000ATB	Statin
atorvastatin	111502AT B	Statin	rosuvastatin and telmisartan	630000ATB	Statin
atorvastatin	111503AT B	Statin	rosuvastatin and telmisartan	629900ATB	Statin
atorvastatin	502201AT B	Statin	rosuvastatin and telmisartan	631600ATB	Statin
atorvastatin	502202AT B	Statin	rosuvastatin and telmisartan	630200ATB	Statin
atorvastatin	502203AT B	Statin	rosuvastatin and telmisartan	630100ATB	Statin
atorvastatin	111504AT B	Statin	rosuvastatin and telmisartan	631700ATB	Statin
atorvastatin	502204AT B	Statin	ezetimibe	462201ATB	ezetimibe
rosuvastatin	454001AT B	Statin	simvastatin and ezetimibe	471000ATB	statin and ezetimibe

rosuvastatin	454002ATB	Statin	simvastatin and ezetimibe	471100ATB	statin and ezetimibe
rosuvastatin	454003ATB	Statin	simvastatin and ezetimibe	507800ATB	statin and ezetimibe
pitavastatin	470902ATB	Statin	simvastatin and ezetimibe	553700ATB	statin and ezetimibe
pitavastatin	470901ATB	Statin	atorvastatin and ezetimibe	633900ATB	statin and ezetimibe
pitavastatin	470903ATB	Statin	atorvastatin and ezetimibe	634800ATB	statin and ezetimibe
atorvastatin and amlodipine	472300ATB	Statin	atorvastatin and ezetimibe	634600ATB	statin and ezetimibe
atorvastatin and amlodipine	614500ATB	Statin	atorvastatin and ezetimibe	633800ATB	statin and ezetimibe
atorvastatin and amlodipine	472400ATB	Statin	rosuvastatin and ezetimibe	640800ATB	statin and ezetimibe
atorvastatin and amlodipine	518900ATB	Statin	rosuvastatin and ezetimibe	640900ATB	statin and ezetimibe
atorvastatin and amlodipine	472500ATB	Statin	rosuvastatin and ezetimibe	640700ATB	statin and ezetimibe
rosuvastatin and valsartan	525100ATB	Statin	pravastatin and fenofibrate	519300ACH	statin and fenofibrate
rosuvastatin and valsartan	525000ATB	Statin	simvastatin and fenofibrate	631400ATB	statin and fenofibrate
rosuvastatin and valsartan	525300ATB	Statin	simvastatin and fenofibrate	631500ATB	statin and fenofibrate
