# Supplementary material for: Importance of small vessel disease as a possible cause of sudden sensorineural hearing loss
Source: PLoS One. 2024 May 7;19(5):e0302447. doi: 10.1371/journal.pone.0302447 (PMC11075872; doi:10.1371/journal.pone.0302447)
Supplement: S4 Table — (PDF) [file pone.0302447.s004.pdf]

S4 Table. Procedure code

Treatment	Procedure				Explain
Brain imaging (CT)	HA441	HA471	HA521	HA561	Computed Tomography (Head)
	HA451	HA481	HA531	HA851	
	HA461	HA511	HA551		
Brain imaging (MRA)	HE135	HI135	HJ135	HJ635	Magnetic Resonance Angiography (Cerebrovascular)
	HE235	HI235	HJ235	HJ735	
	HE535	HI535	HJ535		
Brain imaging (MRI)	HE101	HE401	HI301	HJ401	Magnetic Resonance Imaging (Brain, Hippocampus)
	HE102	HE402	HI401	HJ501	
	HE201	HE501	HI501	HJ601	
	HE202	HE502	HJ101	HJ701	
	HE301	HI101	HJ201		
	HE302	HI201	HJ301		
Fundus photography	E6660	E6674	E6681	EZ795	Fundus Autofluorescence, Field Fluorescein Angiography
	E6670	E6675	E6682		
Dialysis	O7020				Hemodialysis
Peripheral artery disease	M6593	M6632	O1643	O2068	Artery Bypass Surgery, Endovascular procedure, Angioplasty, Thrombectomy
	M6594	O0161	O1644	OA632	
	M6595	O0162	O1645	OA633	
	M6596	O0163	O1646	OA636	
	M6597	O0164	O1950	OA637	
	M6599	O0165	O2054	OA638	
	M6601	O0166	O2055	OA639	
	M6602	O0167	O2056	OB632	
	M6603	O0168	O2058	OB633	
	M6604	O0169	O2059	OB636	
	M6605	O0170	O2064	OB637	
	M6611	O0171	O2065	OB638	
	M6612	O0226	O2066	OB639	
	M6613	O0227	O2067		
Coronary revascularization	HA670	M6563	O1830	OA631	Coronary angiography, Artery Bypass Surgery, Endarterectomy, Thrombectomy, Angioplasty
	HA680	M6564	OA641	OA634	
	HA681	M6571	OA642	OA635	
	HA682	M6572	OA647	OB631	
	M6551	M6634	M6620	OB634	
	M6552	O1641	M6633	OB635	
	M6561	O1642	O2053		
	M6562	O1647	O2057		
