# Supplementary material for: Importance of small vessel disease as a possible cause of sudden sensorineural hearing loss
Source: PLoS One. 2024 May 7;19(5):e0302447. doi: 10.1371/journal.pone.0302447 (PMC11075872; doi:10.1371/journal.pone.0302447)
Supplement: S5 Table — (PDF) [file pone.0302447.s005.pdf]

S5 Table. Drug code & Procedure code in Sudden sensorineural hearing loss

	SSNHL				Explain
Diagnosis	H91.2				ICD-10
Drug code	141901ATB	142030BIJ	217034ASY	193601BIJ	Steroid (Prednisone, methylprednisolone)
	141903ATB	142230BIJ	217035ASY	193603BIJ	
	142232BIJ	217001ATB	193302ATB	193604BIJ	
Procedure code	E6931	E6935	F6341	F6345	Pure tone audiometry
	E6932	E6936	F6342	F6346	
	E6933	E6937	F6343	F6347	
	E6934	F6340	F6344	F6348	
